# Parasites of domestic owned cats in Europe: co-infestations and risk factors

**DOI:** 10.1186/1756-3305-7-291

**Published:** 2014-06-25

**Authors:** Frédéric Beugnet, Patrick Bourdeau, Karine Chalvet-Monfray, Vasile Cozma, Robert Farkas, Jacques Guillot, Lénaïg Halos, Anja Joachim, Bertrand Losson, Guadalupe Miró, Domenico Otranto, Marine Renaud, Laura Rinaldi

**Affiliations:** 1 Merial S.A.S., 29 Av Tony Garnier, Lyon 69007, France; 2 VetAgro Sup, Campus Vétérinaire de Lyon, Marcy-L’Étoile, Lyon, France; 3 Oniris, Site de la Chantrerie, Atlanpôle, Nantes, France; 4 University of Agricultural Sciences and Veterinary, Faculty of Veterinary Medicine, Medicine, Cluj-Napoca, Romania; 5 Faculty of Veterinary Science, Budapest, Hungary; 6 Ecole Nationale Vétérinaire d’Alfort, Parasitology, Maisons-Alfort, France; 7 Department of Pathobiology, Institute of Parasitology, University of Veterinary Medicine Vienna, Wien, Austria; 8 Ecole Vétérinaire, Liège, Belgium; 9 Veterinary Faculty, Universidad Complutense de Madrid, Madrid, Spain; 10 Veterinary Faculty, Bari, Italy; 11 Veterinary Faculty, Napoli, Italy

**Keywords:** Cats, Ectoparasites, Endoparasites, *Toxocara cati*, *Ctenocephalides*, *Otodectes*, Europe, Risk factors, Treatments

## Abstract

**Background:**

Domestic cats can be infested by a large range of parasite species. Parasitic infestations may cause very different clinical signs. Endoparasites and ectoparasites are rarely explored in the same study and therefore multiparasitism is poorly documented. The present survey aimed to improve knowledge of the prevalence and risk factors associated with ecto- and endoparasite infestations in owned cats in Europe.

**Methods:**

From March 2012 to May 2013, 1519 owned cats were included in a multicenter study conducted in 9 veterinary faculties throughout Europe (Austria, Belgium, France, Hungary, Italy, Romania and Spain). For each cat, ectoparasites were checked by combing of the coat surface associated with otoscopic evaluation and microscopy on cerumen samples. Endoparasites were identified by standard coproscopical examinations performed on fresh faecal samples. Risk factors and their influence on parasitism were evaluated by univariate analysis followed by a multivariate statistical analysis (including center of examination, age, outdoor access, multipet status, and frequency of treatments as main criteria) with logistic regression models.

**Results:**

Overall, 50.7% of cats resulted positive for at least one internal or one external parasite species. Ectoparasites were found in 29.6% of cats (CI_95_ 27.3-32.0%). *Otodectes cynotis* was the most frequently identified species (17.4%), followed by fleas (15.5%). Endoparasites were identified in 35.1% of the cats (CI_95_ 32.7-35.7%), including gastro-intestinal helminths in 25.7% (CI_95_ 23.5-28.0), respiratory nematodes in 5.5% (CI_95_ 4.2-7.0%) and protozoans in 13.5% (CI_95_ 11.8-15.3%). *Toxocara cati* was the most commonly diagnosed endoparasite (19.7%, CI_95_ 17.8-21.8%). Co-infestation with endoparasites and ectoparasites was found in 14.0% of the cats, and 11.9% harbored both ectoparasites and gastro-intestinal helminths.

Age, outdoor access, living with other pets, and anthelmintic or insecticide treatments were significantly associated with the prevalence of various parasites.

**Conclusions:**

This survey demonstrates that parasitism is not a rare event in European owned cat populations. The prevalence of multi-parasitism is significantly greater than expected by chance and hence there is tendency for some individual cats to be more prone to infestation by both endo- and ectoparasites due to common risk factors.

## Background

In Europe, domestic cats can be infested by a wide range of endo- and ectoparasites. Depending on the parasite species and its abundance, infestations may cause varying clinical signs in cats, from mild gastro-intestinal disorders and failure to thrive, to anemia or anorexia in the more severe cases, particularly in kittens with heavy parasitic burdens [1]. In addition, some parasites of cats have a zoonotic potential, either through close contact with parasitized animals or through exposure to a contaminated environment [2-6]. This is the case for some nematodes such as *Toxocara cati* and *Ancylostoma tubaeformae*, which are responsible for human visceral/ocular and cutaneous larva migrans, respectively [3-5]. Humans may also become infested with zoonotic cestodes from cats such as *Dipylidium caninum* or *Echinococcus multilocularis*[4-6]. Amongst protozoans, *Toxoplasma gondii* is of major importance in public health, nevertheless, it is recognized that the main source of infection for humans is related to consumption of meat and less to the occysts [7-9]. Ectoparasites can cause direct damage when infesting pets, such as discomfort, pruritus and allergic reactions, but they have also a potential vectorial role: fleas are for instance involved in the transmission of zoonotic pathogens, especially *Bartonella henselae*, the causative agent of cat-scratch disease [10,11].

Parasites of cats are thus a threat for both animal and human health. However, there is much less data available on parasitism in cats compared to dogs.

Overall, the prevalence of endoparasites of cats in Europe has been found to vary between 20 and 40% [12-18]. Almost all surveys carried out so far have been based on coproscopical analysis and focused on the carriage of intestinal nematodes, cestodes and protozoans. The prevalence appears to be higher in cats from shelters or in stray cats, varying from 33% to 90-100% in some studies [14,19,20]. However, these estimates depend on the sample size and the diagnostic procedure used. *Toxocara cati* is usually the most common helminth diagnosed, with infestation rates ranging from 4% to 35% [12-18,20-24]. The other species of ascarids affecting cats, *Toxascaris leonina* is generally found with considerably lower prevalence, rarely exceeding 1% [15,18,22,24,25].

The prevalence of *A. tubaeformae* was found to range from 0.3-0.4% [15,16,22] to around 1% [13,14,18,25]. Higher prevalence rates were found in Romania (10.1%) [17], in Hungary (11.1%) [12] and in Portugal (19.1%) [19].

Tapeworms are rarely found during general endoparasite faecal examinations, but this low prevalence is likely to be related to the poor sensitivity of coproscopy for the detection of cestodes [26]. Indeed, based on coproscopy, prevalence rates for Taeniidae generally do not exceed 5% throughout Europe [12,14,15,17,19,23,25], while the frequency of *D. caninum* infestation has been found to range from 0.1% to 1% [13,15-17].

Based on coproscopical examination, around 1% of cats have been estimated to shed *Giardia* cysts [14,17]. However, higher prevalence rates of *Giardia* have been recorded when using copro-antigen ELISA: 12.6% in Germany [15], 27.9% in Romania [27] or 37.4% in Hungary [12]. This is supported by the overall prevalence of *Giardia* (20.3%) recorded in a 2005–2006 survey on 4214 cats conducted in 7 European countries (most of the samples coming from Germany, Spain and Italy) [28].

Few data are available on the prevalence of coccidia in cats in Europe. Figures for *Cystoisospora* spp. ranged usually between 4 to 8% [12-15,17,29,30], whereas much higher prevalence infestations were recorded in Germany (i.e. 7 to 11%) [14,25] and up to 46.3% for *C. rivolta* in Portugal in stray cats [19]. Concerning *Toxoplasma gondii*, the oocyst shedding is considered to be very low in owned cats. The most recent study, including 24106 cats, found 0.11% of cats with *Toxoplasma gondii* oocyst, and 0.09% for *Hammondia* (final diagnosed through PCR-RFLP) [9].

The cat lungworm, *Aelurostrongylus abstrusus*, seems to be more common than might be expected. In previous records from endoparasite surveys, estimated prevalence in Europe varied between 0.5% and 3% [10-13,15,21,23] and infestation was considered to be sporadic. Studies reported either isolated clinical cases [31] or the finding of larvae in fecal examinations performed on symptomatic cats. In Germany, two recent studies established infestation rates of about 6% in symptomatic cats [32,33]. The distribution of this parasite seems to be spreading in several countries, with prevalence rates up to 20% in enzootic areas [34]. This is especially the case in Italy, with reported infestation rates of 8.5% [35] and 17.6% [36] or in Portugal with 12.4% and 17.4% positive cats in recent surveys [19,37]. The highest rate (43.1% of 58 fecal samples) was found in Tirana area, Albania [38]. Other metastrongyloids such as *Troglostrongylus brevior* and *Troglostrongylus subcrenatus* have also been recently reported as causative agents of respiratory infestation in domestic cats in Spain and Italy [39-41]. However, information concerning the impact of this species of lungworm on feline populations is scarce and limited to a few case reports and it is currently unclear whether their occurrence in domestic cats is sporadic, neglected or underestimated [42,43]. In a recent epidemiological survey carried out in Sardinia (Italy), the 29.9% (32/107) of examined cats were infested by broncho-pulmonary nematodes and, although *A. abstrusus* was the most frequently detected (n = 27; 25.2%), larvae of *T. brevior* were found in 6.5% (7/107) of samples with two cats (1.9%) being co-infected by both species [44]. Some other respiratory nematodes may be found by coproscopy techniques, especially *Capillaria*, with a reported prevalence around 1 to 5% [12-15,17,18].

Fleas are a common ectoparasite of cats, with prevalence ranging from 12% up to 70% [12,45-47]. In some countries, infestation rates of more than 70% have been observed (Spain, Germany, Austria) [48]. Seasonal variations are observed, with a lower prevalence in winter (12%) in comparison with spring or summer (21%) [46]. Other factors such as the habitat (rural or urban), the presence of other animals in the household the use or not of flea control are also known to influence the prevalence of fleas [46,47]. Moreover, fleas are more common in multi-pet households [45]. The use of a flea control product also plays a major role in the occurrence of flea infestation, as more than half (51.4%) of the infested cats were not treated with an ectoparasiticide over the previous year [47]. Three species of fleas are commonly identified in cats: *Ctenocephalides felis*, *C. canis* and *Pulex irritans*, with a preponderance of *C. felis felis*, which is identified in almost all flea-infested cats [12,45-47,49].

Data for tick infestation in cats is rare. In a recent survey in Belgium, *Ixodes ricinus* was found in 80.1% of the tick-infested cats and the only other tick species found was *I. hexagonus* (23.4%) [50]. A survey conducted in the South of France revealed that 30% of cats coming for veterinary consultation were infested with ticks belonging to the *Ixodes* and *Rhipicephalus* genera [51].

Infestation by the ear mite *Otodectes cynotis* may be locally frequent in cats in some areas of Europe, especially in kittens and roaming animals. In a survey conducted in Greece, 25.5% of owned cats resulted positive, with age identified as a risk factor [52] and 14% of kittens up to 6 months from urban areas and without external otitis signs [53]. In another survey conducted in Italy, *O. cynotis* was identified as the primary cause of external otitis in 53.3% of 1087 stray cats examined [54].

No previous study explored endoparasites and ectoparasites in the same animal hosts and therefore multiparasitism is poorly documented. The present survey aimed to improve our knowledge on the prevalence of the occurrence of ecto- and endoparasite infections, in household owned cats in Europe and to examine risk factors and their influence on parasitism.

## Methods

### Animal selection

From March 2012 to May 2013, a multicenter survey was conducted throughout Europe for parasite infestations in client-owned household cats. Nine veterinary faculties participated: 2 in France (Maisons-Alfort, Nantes), 2 in Italy (Bari, Naples) and 1 each in Austria (Vienna), Belgium (Liège), Hungary (Budapest), Romania (Cluj-Napoca) and Spain (Madrid).

A random sample of cats was included weekly (with an objective of 10 to 20 cats per week) from the consultation services in the Faculty hospitals. Client-owned cats were eligible for participation in the study provided that they were in good health, and were not presented for a medical reason related to any parasitic disease. In addition, cats should not have received an anthelmintic treatment for two months prior to inclusion nor an ectoparasiticide for one month prior to inclusion. Cats that were sick or were difficult to handle for parasite comb counting were excluded from the study. Details of each enrolled cat including demographic data, household accommodation, hunting behaviour and previous parasiticide treatments were recorded on an owner questionnaire form.

### Methodology for parasite detection and identification

An informed consent and agreement was obtained from the owners of the cats before enrolment. The examination of cats were conducted with regard to animal welfare.

Cats were combed for at least 7 minutes and combing continued until no further parasites had been removed for 3 consecutive minutes. The collected fleas or ticks were stored in individual vials containing 60% ethanol for identification of species. The external ear canals of all cats were examined and cerumen sampled for microscopical search of *Otodectes*. Cats with suspected skin mite infestations were assessed by skin scraping and microscopical observation.

The detection of endoparasites was based on qualitative coprological analyses performed by trained people in expert centers. The techniques and protocol used in each laboratory were those routinely conducted for the diagnosis of parasitical infestation in animals and in accordance with the standard guidelines for parasitological diagnosis. Slight variations in the techniques and protocols used was observed from one laboratory to another. No quantitive analysis was requested and all techniques were considered as reasonably sensitive for an accurate detection (range from 2 to 6 eggs/cysts per gram of faeces) making it possible to rely on the global results and compare results [55-57].

Faecal samples were collected from all cats at the time of consultation or by the owner within 24 hours. Both macroscopic examination (i.e., to identify adult parasites and cestode proglottids) and microscopic examination (i.e., to identify parasite oocysts, cysts, eggs, and larvae), were performed. In all laboratories, direct centrifugal flotation techniques were used [55-57]. They were all derived from the Stoll 1923 (modified 1930) method. The flotation liquid used was: Zinc sulphate (s.g. 1.18 or s.g. 1.35), Zinc Sulphate + Acetate (s.g. 1.33), Sodium chloride (s.g. 1.2), and Sucrose solution (1.20). One center used the FLOTAC device [57] whilst the others used classical centrifugation (1 min, 1500 rpm) in tubes. The ratio between flotation liquid and faeces quantity was 1/12 to 1/15. In 3 faculties (Spain, Austria and France) an additional initial centrifugation step either in water or in acetic acid (Telemann method) was added. In addition, Baermann technics were run in Italy, Hungary and Romania.

All data collected during the study were uploaded electronically to a secure dedicated on line web database.

### Statistical analysis

Prevalence rates were recorded as the ratio of the number of positive animals to the total number of examined animals. The 95% confidence intervals were computed with R software [58], by means of exact Clopper–Pearson method. Association between parasitism and risk factors was first screened by univariate analysis (contingency tables and χ² (chi-squared tests)) and then by multivariate analysis with binary logistic multiple-regression [59]. The dependent variable was set as the binary outcome infestation/non infestation, while the explanatory variables were: center of examination (Maisons-Alfort, Bari, Budapest, Cluj-Napoca, Liege, Madrid, Nantes, Naples, Vienna), age of cats (i.e., < 6 months, 6 months to 2 years, and > 2 years), outdoor access (i.e., infrequent or frequent), multi-pet household (i.e., single animal or multi-pet household), number of other cats in the house (i.e., no other cat, 1 or 2 other cats, more than three other cats), frequency of anthelmintic treatment per year (3 modalities, 0, 1 or 2, and ≥ 3 treatments per year) and frequency of ectoparasiticide treatment (≤3 treatments, ≥ 4 treatments). The first two age-classes were chosen to include enough cats to provide the best statistical power. For the multivariate analysis, the reference for the center of examination was set as Hungary, given the reliable number of animals included (300) and the average results they obtained for the analyzed parasites when compared with the other centers. The references for other variables were: cats from 6 to 24 months old, cats with frequent outdoor access, cats living with other pets, cats living with 1 or 2 other cats, cats receiving 1 or 2 anthelmintic treatments per year and cats receiving less than 3 ectoparasiticide treatments per year.

The model building strategy was that all the factors and their likely interactions entered the logistic regression. The model was then reduced with the function stepAIC in R MASS package for non-nested models. Significance of variables was evaluated, one at a time, by Likelihood Ratio Tests to compare the deviances of nested models. If non-significant, backward eliminations were performed until the most parsimonious model was fitted.

The goodness of data fit to the model was assessed graphically by plotting the standard residuals, and by computing the pseudo R-squared (McFadden). Hosmer-Le-Cessie tests or Hosmer-Lemeshow tests were also performed to assess the model. The odds ratios (OR) and their 95% CIs were calculated by exponentiation of the estimates. Significance was set at p < 0.05.

## Results

### Demographic data

A total of 1519 client-owned cats were recruited: 92 in Vienna (Austria), 55 in Liege (Belgium), 96 in Maisons-Alfort (France), 91 in Nantes (France), 300 in Budapest (Hungary), 300 in Bari (Italy), 215 in Naples (Italy), 300 in Cluj-Napoca (Romania) and 70 in Madrid (Spain). Of the 1519 cats, 57.0% (866) were female (46.5% entire and 53.5% neutered) while 43.0% (653) were male (52.8% entire and 42.2% castrated).

Age data were available for 1500 of the cats, ranging from 1 month to 22 years and 5 months, with a mean of 3 years and 5 months and a median of 2 years and 7 months: 223 cats (14.9%) were less than 6 months, 470 cats (31.3%) were between 6 months and 2 years old, and 807 cats (53.8%) were over 2 years old. A minority of cats had infrequent access to the outdoors, whereas 1093 cats (72.0%) had frequent access to the outdoors, including 558 (51.1%) having unlimited access to the outdoors (all the surrounding environment) and 484 (44.3%) having limited access to the outdoors (closed garden).

A total of 1147 cats (75.5%) lived in multi-animal households. Among animals living in multi-animal households, 1021 (89.0%) lived with at least one other cat, while 480 (41.8%) lived with at least one dog and 385 (26.0%) lived with at least one cat and one dog. The number of cats living in a house was reported as 1481 cats: 460 cats (31.0%) lived alone (no other cats), while 444 cats (30.0%) lived with 1 or 2 other cats and 577 cats (39.0%) lived with more than 3 other cats.

The number of ectoparasiticide treatments given per year was available for 1379 of the cats. This ranged from 0 to 12, with 1131 cats (82.0%) treated less than 3 times a year, and 248 cats (18.0%) treated 4 times or more per year.

The number of endoparasiticide treatments given per year was available for 1256 of the cats. This ranged from 0 to 12, with 0 treatments per year for 430 cats (34.2%), 1 or 2 treatment per year for 653 cats (52.0%) and more than 3 treatments per year for 173 cats (13.8%).

**Figure 1:**
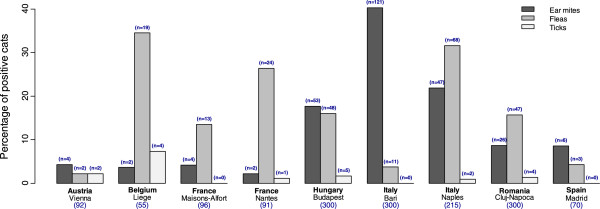
Prevalence of infestation with ectoparasites per country (and number of cats infested).

**Figure 2:**
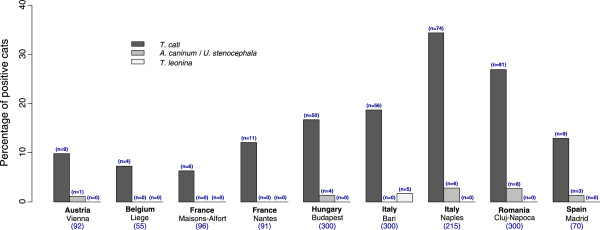
Prevalence of infestation with gastro-intestinal nematodes per country (and number of cats infested).

**Figure 3:**
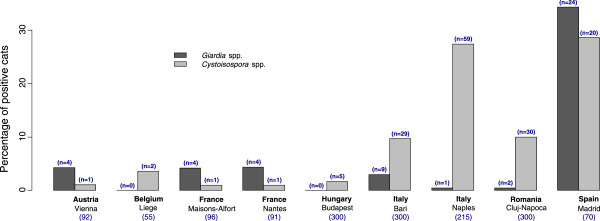
Prevalence of infestation with gastro-intestinal protozoans per country (and number of cats infested).

A total of 50.7% (770/1519) of cats examined in this study were positive for parasites (Table 1, Figures 1, 2 and 3).

**Table 1:** Observed prevalence of intestinal parasites (as determined by standard coproscopic examinations)

	**% (n)**	**95% confidence interval**
**Gastro-intestinal nematodes**	20.5% (312*)	19.1 – 23.3
*T. cati*	19.7% (300*)	17.8 – 21.8
*A. tubaeforme*/*U. stenocephala*	1.4% (22*)	0.9 – 2.2
*T. leonina*	0.3% (5*)	0.1 – 0.8
**Gastro-intestinal cestodes**	7.0% (107*)	5.8 – 8.4
*D. caninum*	3.0% (45*)	2.2 – 3.9
*Taeniidae*	1.3% (19*)	0.8 – 1.9
**Respiratory nematodes**	5.4% (61**)	4.2 – 7.0
*A. abstrusus*	4.1% (46**)	3.0 – 5.5
*Capillaria* spp.	1.1% (16*)	0.6 – 1.6
**Protozoans**	13.5% (205*)	11.8 – 15.3
*Giardia* spp.	3.2% (48*)	2.3 – 4.2
*Cystoisospora* spp.	9.7% (148*)	8.3 – 11.3
**Co-infestations**		
External + internal parasites	14.0% (213*)	12.3 – 15.9
External parasites + gastro-intestinal helminthes	11.9% (181*)	10.3 – 13.7
Fleas + gastro-intestinal nematodes	5.4% (82*)	4.3 – 6.7
Fleas + *T. cati*	5.3% (80*)	4.2 – 6.5

*(N) number of positive cats for the criteria out of 1519 cats.

**(N) number of positive cats for the criteria out of 1115 cats.

### Ectoparasites

The overall rate of infestation of cats with ectoparasites was 29.6% (450/1519). Ear mites were identified in 17.4% (265/1519) of examined cats, followed by fleas in 15.5% of cats (235/1519), ticks in 1.2% of cats (18/1519) and other ectoparasites in 1.4% of cats (21/1519). Other ectoparasites reported included *Felicola subrostratus* in 13 cats, *Cheyletiella blakei* in 5 cats and *Notoedres cati* in 3 cats.

### Endoparasites

From macroscopic and microscopic analysis of faecal samples, 35.1% (533/1519) of cats were positive for endoparasites (gastro-intestinal helminths, protozoans or lungworms) (Table 1). Gastro-intestinal helminthosis was found in 25.7% (390/1519) of cats, showing a higher prevalence than protozoan infections recorded in 13.5% (205/1519) of cats or respiratory nematodes in 5.5% (61/1115) of cats.

Gastro-intestinal (GI) nematodes were recorded in 20.5% of the examined cats, including *T. cati* in 19.7%, *A. tubaeforme*/*Uncinaria stenocephala* in 1.4% and *Toxascaris leonina* in 0.3% of cats. Ascarids were seen macroscopically in 6 faecal examinations (0.4%). One cat was found to excrete *Physaloptera* spp., *Capillaria* spp. eggs were identified in 1.0% of the 1519 examined cats.

Cestode eggs were detected in 7.0% of cats, including *Dipylidium caninum* in 3.0% and Taeniidae in 1.3% of cats. Cestode proglottids were also observed macroscopically in 52 faecal samples (3.4%).

GI protozoans were found in 13.5% of cats: 9.7% and 3.2% of coproscopic samples were positive for *Cystoisospora* spp. and *Giardia* spp., respectively. Other flagellates were found in 4 cats (0.3%), and other coccidians in 9 cats (0.6%). No *Toxoplasma* like oocyst was observed during this survey.

The Baermann technique, recommended to separate nematode larvae from faecal material, was not performed in all the examination centers. For the four centers of Bari, Budapest, Cluj-Napoca and Naples having performed this specific search, *A. abstrusus* was identified in 4.1% of the 1115 examined cats.

### Analysis of risk factors

Risk factors were specifically analysed only when the rate values made it possible. Therefore, specific analysis was possible for *T. cati*, *A. abstrusus*, *C. felis *and *O. cynotis*.

### Co-infestations

A total of 305 cats (20.1%) harboured more than two parasite species, and 147 cats (9.7%) harboured more than two endoparasite species, while 14.0% (95% CI 12.3-15.9) of cats were found infested with both external and internal parasites (Table 1). By chance, the prevalence of ecto + endoparasite infestation should be estimated as 10.4% (29.6% for ectoparasites * 35.1% for endoparasites). Both prop-test and Fisher test confirmed that the observation is significantly different than the pure random (p < 0.01). A χ² test confirmed that endoparasite and ectoparasite infestations were dependant variables (p-value < 10^−9^), and the number of cats parasitized with both endo and ecto-parasites (213) was more important than expected by chance (158). Infestation with ectoparasites and GI helminths was found in 11.9% (181/1519) of cats while 5.3% (81/1519) harboured both fleas and *T. cati*. Computed odds ratios for the presence of an external parasite as a risk factor for endoparasite infestation showed that cats harboring external parasites (ear mite, fleas or other parasites) had a significantly higher risk to also harbor endoparasites (respiratory nematodes, gastro-intestinal nematodes, cestodes or protozoans) (OR 2.10, CI_95_ 1.68-2.64).

### Risk factors for *Toxocara cati* infestation

**Figure 4:**
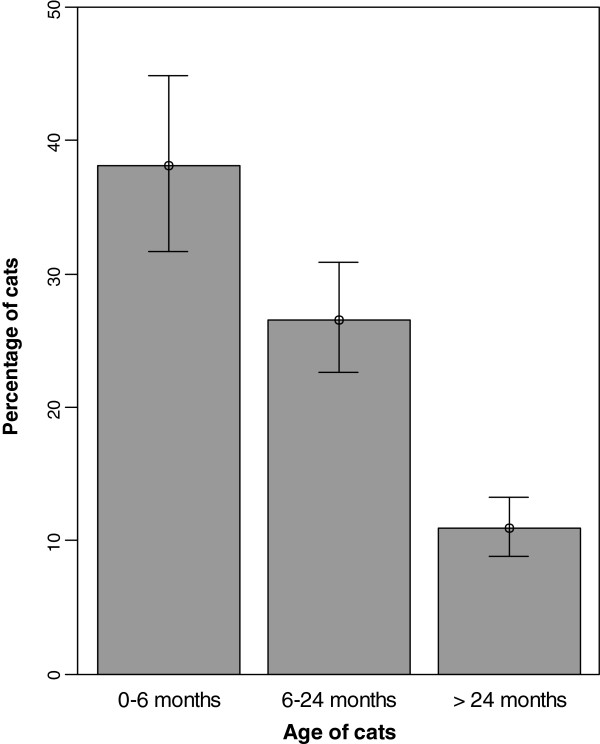
**Influence of the age on *T. cati*** **estimated prevalence, with 95% CI (represented by the whiskers).**

**Figure 5:**
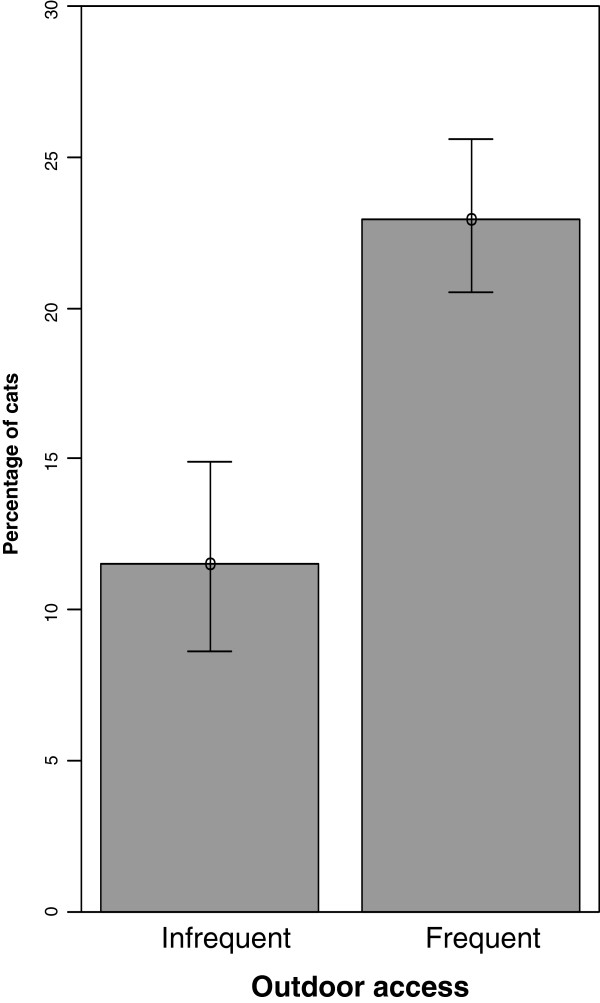
**Influence of the frequency of treatment against *Toxocara, *** **with 95% CI (represented by the whiskers).**

From univariate analysis (contingency tables and χ² tests), significant correlations (p < 0.05) were found between *T. cati* infestation and the following criteria: age, outdoor access, frequency of anthelmintic treatment, number of other cats in the house, center of examination (Table 2, Figures 4 and 5).

**Table 2:** **Significance of risk factors with *Toxocara cati *** **infestation**

**Variables**	**% (n) of cats postive for** ***T. cati***	**χ² (chi-squared tests)**
**Origin**		
Alfort	6.3% (6)	p < 10^−11^
Liege	7.3% (4)	
Vienna	9.8% (9)	
Nantes	12.1% (11)	
Madrid	12.9% (9)	
Budapest	16.7% (50)	
Bari	18.7% (56)	
Cluj-Napoca	27.0% (81)	
Naples	34.4% (74)	
**Age**		
<6 months	38.1% (85)	P < 10^−15^
6-24 months	26.6% (125)	
>24 months	10.9% (88)	
**Outdoor access**		
Rare	11.5% (49)	P < 10^−6^
Frequent	23.0% (251)	
**Number of anthelmintic treatment per year**		
0	23.3% (100)	P = 0.001
1 or 2	22.2% (145)	
More than 3	10.4% (18)	
**Number of other cats in the house**		
0	17.6% (81)	P = 0.001
1 or 2	15.5% (69)	
3 or more	24.1% (139)	

All these significant risk factors as determined from univariate analysis were entered in the multiple logistic regression model, to address possible confounding factors and to compute adjusted odds ratios (Table 3).

**Table 3:** **Adjusted odds ratios CI**_**95**_**and significance of the p-value (Wald Test) from logistic regression models**

	***Toxocara cati***			***Aelurostrongylus abstrusus***		***Otodectes cynotis***		***Ctenocephalides spp.***			
**Center of examination**											
Maisons-Alfort	0.66 [0.23-1.62]		NS			0.30 [0.08-0.82]	*	2.10 [0.97-4.38]	NS (.)		
Bari	2.51 [1.29-4.82]		**	0.64 [0.20-1.84]	NS	4.06 [2.75-6.06]	*	0.19 [0.07-0.43]	***		
**Budapest**	**1.00**		**-**	**1.00**	**-**	**1.00**	**-**	**1.00**	**-**		
Cluj-Napoca	2.81 [1.82-4.39]		***	1.56 [0.64-3.83]	NS	0.45 [0.25-0.76]	**	1.43 [0.90-2.27]	NS		
Liege	0.42 [0.12-1.13]		NS			0.22 [0.03-0.74]	*	5.30 [2.60-10.66]	***		
Madrid	0.75 [0.24-1.97]		NS			0.53 [0.19-1.24]		0.64 [0.13-0.63]	NS		
Nantes	1.30 [0.52-2.95]		NS			0.15 [0.02-0.52]	*	4.86 [2.46-9.51]	***		
Naples	5.05 [3.07-8.43]		***	4.26 [1.96-9.81]	***	1.73 [1.09-2.74]	*	7.69 [4.60-13.02]	***		
Vienna	0.79 [0.28-1.88]		NS			0.23 [0.07-0.59]	**	-	-		
Age of cats											
<6 months	2.12 [1.32-3.44]		**	-	-	0.72 [0.40-1.25]	NS	1.09 [0.62-1.93]	NS		
**6-24 months**	**1.00**		**-**	**-**	**-**	**1.00**	**-**	**1.00**	**-**		
>24 months	0.64 [0.43-0.93]		*	-	-	0.99 [0.70-1.42]	NS	1.50 [0.99-2.31]	NS		
Outdoor access											
Rare	0.41 [0.21-0.75]		**	0.22 [0.06-0.55]	**	0.35 [0.23-0.53]	***	0.26 [0.17-0.41]	***		
**Frequent**	**1.00**		**-**	**1.00**	**-**	**1.00**	**-**	**1.00**	**-**		
Multipet household	Number of other cats in the house					Multipet household					
	0	1.34 [0.89-2.01]	NS			No	1.11 [0.56-2.09]	*	No	1.25 [0.64-2.36]	NS
	**1-2**	**1.00**	**-**			**Yes**	**1.00**	**-**	**Yes**	**1.00**	**-**
	≥ 3	2.27 [1.54-3.36]	***								
Treatments	Anthelmintic treatment per year (nb)								Anti-flea treatment per year (nb)		
	0	1.54 [1.05-2.27]	*						**≤3**	**1.00**	**-**
	**1-2**	**1.00**	**-**						≥4	0.22 [0.13-0.37]	***
	≥ 3	10.48 [0.27-0.83]	*								
Interactions	>24 months + rare outdoor access					<6 months + single animal			>24 months + single animal		
	0.30 [0.10-0.81]		*			5.36 [1.92-15.23]		**	0.40 [0.17-0.92]		*

***0 < p < 0.001.

**0.001 < p < 0.01.

*0.01 < p < 0.05.

NS 0.05 < p.

(.) 0.05 < p < 0.1.

The modalities taken as references for the statistical analysis are presented in bold.

With an age category of 6–24 months (26.6% positive cats) as a reference, cats older than 24 months (10.9% positive) were at lower risk of being infested with *T. cati* (adjusted OR 0.64, 95% CI 0.43-0.93), while cats under 6 months (38.1% positive) were at higher risk (adjusted OR 2.12, CI_95_ 1.32-3.44).

Cats with infrequent outdoor access (11.5% positive) were significantly less frequently infested with *T. cati* than cats having frequent access (23.0% positive) to the outdoors (adjusted OR 0.41, CI_95_ 0.21-0.75).

Compared to the reference center of Budapest (16.7% positive), Naples (34.4%) and Bari (18.7%) presented significant higher frequencies of infestation after adjustment of putative confounding factors (age, outdoor access, multipet household and frequency of anthelmintic treatments). The following examination centers showed no significant differences: Maisons-Alfort (6.3%), Vienna (9.8%), Liège (7.3%), Nantes (12.1%) and Madrid (12.9%).

The risk of being *Toxocara* positive was significantly higher for cats from households with more than 3 cats (24.1% positive, adjusted OR 2.27, CI_95_ 1.54-3.36).

Regarding the frequency of anthelmintic treatments, cats receiving no treatment were significantly more at risk of *Toxocara* infestation (23.3%) than cats receiving 1 or 2 treatments per year (22.2%, adjusted OR 1.54, CI_95_ 1.05-2.27), while cats receiving more than 3 treatments per year were significantly less infested (10.4%, adjusted OR 0.48, CI_95_ 0.27-0.83) than the previous group.

Cats over 24 months old and with infrequent outdoor access were significantly less at risk of *T. cati* infestation than the average (OR = 0.30, CI_95_ 0.10-0.81).

### Risk factors for *Aelurostrongylus abstrusus* infestation

From univariate analysis, significant correlations were found between outdoor access (p = 0.018), location (p <0.001) and *A. abstrusus* infestation. Age was not significantly related to lungworm infestation.

The risk factors were included all together in a logistic regression model (Table 3). Age was not found to be a significant factor. Cats from the Naples center (9.3% positive) were significantly more often infested than cats from the reference center of Budapest (3.3% positive), while Bari (1.7% positive) and Cluj-Napoca (3.7% positive) centers did not show significant differences. Cats with infrequent outdoor access (1.5% positive) were significantly less at risk than cats with frequent access to the outdoors (5.0% positive) (adjusted OR 0.22, CI_95_ 0.06-0.55).

### Risk factors for *Otodectes cynotis* infestation

From the univariate analysis, significant correlations were found between ear mite infestation and outdoor access (p < 10^−6^), multipet household (p <0.001) and location (p <10^−15^). No statistical difference was found at the 5% level for *O. cynotis* infestation with the three age categories. These risk factors were included as explanatory variables in the logistic regression model. In agreement with the results of the univariate analysis, no significant difference was found regarding the risk factor age. Nevertheless, this variable was retained in the regression model since the Likelihood Ratio Tests estimated a significant reduction of the deviance of the model by keeping it (Table 3). After adjustment of counfounding factors, no significant relation was found between the factor multipet household and ear mite infestation, probably due to a decrease in the power of the tests compared to the univariate analysis.

Taking the Budapest center of examination as reference (17.7% positive), cats from the following sites were significantly less infested with ear mites: Maisons-Alfort (4.2%), Vienna (4.3%), Liège (3.6%), Nantes (2.2%) and Cluj-Napoca (8.7%), while cats from Bari (40.3%) and Naples (21.9%) were significantly more infested. Madrid center (8.6%) was not significantly different from Budapest.

Outdoor access was identified as a risk factor, cats with infrequent outdoor access (9.4% positive) being less at risk than cats with frequent outdoor access (20.6% positive) (adjusted OR 0.35, 95% CI 0.23-0.53). Cats under 6 months old and living alone were significantly less at risk than other cats (OR 5.36, CI_95_ 1.92-15.23).

A Hosmer-Lemeshow test was performed to assess the homogeneity between the estimated probabilities and the observed values, and evaluated a correct goodness of fit for the model.

### Risk factors for *Ctenocephalides* spp. infestation

From the univariate analysis (contingency tables and χ² tests), significant correlations (p < 0.05) were found between flea infestation and the location (p <10^−15^) or outdoor access (p < 0.0001). No significant relationship was found between flea infestation and age or multipet household.

These risk factors were entered into a regression model. The Vienna center, due to the low number of animals with fleas (n = 2) was responsible for a poor goodness of fit, and was therefore excluded from the regression model. Following this, cats with frequent outdoor access (18.0% positive) were found to be more at risk for flea infestation than cats with infrequent outdoor access (8.9% positive, adjusted OR 0.26, 95% CI 0.17-0.41). Flea infestation did not differ significantly between cats living alone and cats living with other pets, even after adjustment of confounding factors (adjusted OR 1.25, CI_95_ 0.64-2.36). Nevertheless cats older than 24 months and living in single animal household appeared significantly less at risk for flea infestation (OR 0.40, CI_95_ 0.17-0.92).

Cats treated more than 4 times a year (11.7% positive) were significantly less at risk for flea infestation than cats receiving fewer treatments per year (17.4% positive, adjusted OR 0.22, CI_95_ 0.13-0.37).

When compared with the reference center of Budapest (16.0% positive), cats from the following centers were significantly more frequently infested with fleas: Liege (34.5%), Nantes (26.4%) and Naples (31.6%), while cats from Bari (3.7%) were significantly less frequently infested. The centers of Maisons-Alfort (13.5%), Cluj-Napoca (15.7%) or Madrid (4.3%) did not significantly differ from the Budapest center, after adjustment of confounding factors (i.e. outdoor access, age, treatment frequency).

## Discussion

The present survey offers the first large scale overview on parasite infestation of the European owned cat population. It demonstrates that more than half (50.7%) of the owned cat population carry at least one parasite at a given time with a high level of co-infestations.

Prevalence values in the present survey were estimated for healthy cats that were not presented to veterinary consultation for a medical reason related to parasitic disease. The real prevalence of parasites may be much higher, as many potentially parasitic conditions were excluded from the study. Regarding internal parasites, a study in Spain found a significantly higher prevalence in stray cats (32.9%) than in household cats (16.5%) [18]. Previous surveys conducted in Germany, Italy and Hungary found endoparasites in 22.8%, 35% and 39.6% of owned cats respectively, which is close to our results, i.e. 35.1% [12,13,15].

The observations were performed at a single time-point for each cat, therefore, due to the natural lifecycle of parasites, which include intermittent egg/cyst shedding, this may have underestimated the observed prevalence.

The discrepancies between the different locations may have been related to the fact that different techniques and technicians performed the examinations, with specific local expertise (i.e. detection of *Giardia* cysts). However, this factor was taken into consideration at the setup of the study and several considerations allowed consideration of the comparisons as valid.

Each location performed direct centrifugal flotation methods. One used the FLOTAC device [57], the others classical tubes and rotors. Different but well known flotation liquids were used (specific gravity ranging from 1.18 to 1.28), and the same ratio between faeces and volume (i.e. 1/15). This could be a factor of bias for some eggs i.e. trematode eggs, but the sensitivity is considered similar for the nematode and cestode eggs as well as protozoan cysts. The sensitivity of the FLOTAC device and technique is evaluated at 2 epg compared to 5/6 epg for the others. This could be of importance in comparisons of quantitative coproscopy, which was not the case here, where only qualitative data were searched. Nevertheless, the potential bias due to the center of analysis was, inter alia, one of the issues addressed by the multivariate analysis. The examination center was entered as a confounding factor of bias (random effect), meaning that the bias was mainly due to the methodologies used rather than true differences due to geographical locations.

The overall prevalence of endoparasites found in cats in this study (35.1%) is close to that found in previous epidemiological surveys in Europe [12-15,17]. The infestation rates per parasites are in the range of the literature data described above. A slightly higher rate (4.1%) was found for *Aelurostrongylus abstrusus* infestation when compared to those previously reported in Europe from standard general endoparasite surveys (i.e. less than 3%) [14,18,23,25]. However, according to recent works, Italy would be an endemic area with prevalence rates reaching 20% [34,35] and high rates were also reported recently for Romania (5.6%) [17] and Hungary (14.5%) [12]. It should be noted that *Aelurostrongylus abstrusus* in the present survey may include lungworms of the genus *Troglostrongylus*, due to the morphological similarities of the first stage larvae and the consequently low specificity of diagnosis by non-specialists [43].

No *Toxoplasma gondii* like oocyst was found during this survey. It gives a CI_50_ of 0–0.24%. A coproscopy survey conducted throughout Europe in 2008 found a prevalence of 0.11% for *Toxoplasma* and 0.09% for *Hammondia*, based on 24106 owned cats, which gives 0.20% of *Toxoplasma* like oocysts [9]. The absence of typical oocyst in our 1519 owned cats can be explained by this very low prevalence. We found one 6 month old cat excreting *Toxoplasma* oocysts in a parallel analysis of 153 stray cats in Madrid (unpublished). The infection rate is significantly higher in young cats, stray cats, than owned cats eating dried food, as demonstrated in a serological survey conducted in Portugal [7]. A study conducted in Virginia (USA) highlighted that oocyst shedding appeared one month after infection and lasted around 21 days only, followed by the installation of immunity [8]. These reasons can explain the difficulty to see the Toxoplasma oocysts when doing routine coproscopy.

Mixed infestations with more than two endoparasites were found in 15-18% of cats in Hungary and Romania [12,17] and 28-30% of parasitized cats in Italy and Spain [13,18], to compare to the lower rate (9.7%) obtained here but estimated for owned cats exclusively.

The present survey shows that 14.0% of cats were co-infested with at least an external and an internal ectoparasite (mainly external and gastro-intestinal parasites - 11.9% of cats). The number of cats with both endo-and ecto-parasites (213) was more important than expected by chance (153, p-value of χ² test <10^−9^). This significant association should be related to similar risk factors considering the mode of life of the cats and probably not to a parasitic effect. Moreover, cats with the presence of an external parasite were more likely to harbour internal parasites than cats without ectoparasites (Odds Ratio 2.10 CI_95_ 1.68-2.64).

This supports the fact that an endo and ecto- parasite checking and management should be handled in parallel. The observed co-infestations were mainly with external parasites and gastro-intestinal worms with 11.9% of cats (i.e. protozoans and lungworms excluded), here again significantly higher than expected by chance (p-value of Fisher test < 10^−4^).

Taking into account the poor sensitivity of coproscopy to detect the infestation by *Dipylidium*, co-infestation with fleas and *D. caninum* is not considered as valid in this survey.

Risk factors for infestation with endoparasites have been identified and tested in previous studies [12-15,17]. The associations were usually tested by univariate analysis; with contingency tables and χ^2^−tests or Fisher’s Exact test [12-14]. Some studies performed multivariate analysis [12,14,36,52].

The multivariate analysis, taking into account multiple putative confounding factors, can decrease the power of the statistical tests. Thus, results obtained with univariate analysis may not always be confirmed by the multivariate due to the adjustment of counfounding factors.

As previously observed [12-14], sex did not show any influence on the prevalence of *T. cati*, *A. abstrusus*, *O. cynotis* or fleas.

In several studies, young cats are estimated to be much more frequently infested with digestive parasites especially with ascarids and protozoans (coccidians, *Cystoisospora* spp. and *Giardia*). Meanwhile infestations of hookworms, lungworms, whipworms and taeniid cestodes seem to be mainly prevalent in older cats [13-17]. Regarding *Toxocara* infestation, higher prevalence rates are commonly recorded in young cats, with 26.5% of cats under one year being infested compared to 13.8% for adult ones in a study in Hungary [12]. In agreement with these authors, based on an age category of 6–24 months, cats older than 24 months had a lower risk of being infested with *T. cati* in our survey while cats under 6 months were at higher risk. Nevertheless, it should be highlighted that cats over 6 years where found infested by *T. cati*, meaning that the immunity is not absolute and that veterinarians should not be surprised to see ascarids in adults or even old cats. Hookworms were more prevalent in cats older than 2 years (2.1%, 10/470) than in cats from 6 months to 2 years (1.2%, 10/807), themselves being more infested than cats under 6 months (0.9%, 2/223), however χ² tests performed were not significant due to low frequencies for *Ancylostoma* spp. and *Uncinaria* spp. The age was not a significant risk factor for *A. abstrusus*, *O. cynotis* nor flea infestation, from both univariate and multivariate analysis.

Regarding *A. abstrusus*, this contradicts a multivariate analysis performed in Italy, showing that cats under one year old (24.1% positive) were significantly more at risk than cats over 1 year old (10.8% positive), when adjusted with outdoor access and presence of symptoms [36]. The reason advocated for the effect of age was thought to be the higher preying instinct of young cats.

*Otodectes* infestation was not found to be linked to the age of cats in the present study: indeed, a systematic search was performed, regardless of clinical signs. We can hypothesize that, although otodectic mange may be considered mainly as a kitten-infestation (young cats being the ones developing otitis due to the parasites), adult cats are probably subclinical carriers and reservoirs for other domestic carnivores in the house [60].

Outdoor access was identified as a risk factor in our study for all parasites studied. Cats with infrequent outdoor access were found to be significantly less frequently infested than cats with frequent outdoor access for *T. cati*, *A. abstrusus*, *O. cynotis* and fleas. Outdoor access was also seen to have a significant influence on *T. cati* infestation in previous studies [17]. This may suggest that *T. cati* is not only circulating through a mother-to-kitten transmission during lactation, but that egg persistence in the outdoor environment as well as paratenic hosts play an important role in its transmission [1]. Regarding *A. abstrusus*, Traversa (2008) also found that 38 out of the 40 infested cats had outdoor access, which was estimated as significant after multivariate analysis [36]. This is in accordance with the mode of transmission of this parasite: cats becoming infested by ingesting intermediate or paratenic hosts (rodents, slugs, lizards, frogs).

*T. cati* being a cat-specific parasite, the influence of a multi-cat household as a risk factor was assessed. Cats living with one or two other cats were not significantly more infested than cats living alone, but for higher densities of cat populations (more than 3 other cats in the house), the risk for *Toxocara* infestation was significantly more important. Multi-pet households (this time evaluated regardless to cat or dog co-living) were found to be a significant risk factor for ear mite infestation from univariate analysis with cats living alone (11.6% positive), being less infested than cats living in multipet household (19.4% infested). However, after multivariate analysis, this factor was no longer significant. It can be supposed that the power of the test was decreased due to the other factors. Knowing the biology of the parasite and its direct transmission [60], we can nevertheless consider that living with other pets is indeed a risk factor for ear mite infestation.

Living with other pets was not a relevant risk factor for flea infestation. This could be explained by the fact that close-contact is not needed for flea transmission. The main source of infestation are pupae, which are able to survive for several months in contaminated environments [61]. Therefore, cats living alone in households were not at lower risk for flea infestation.

A recent study in Hungary showed that cats whose owners claim the use of an anthelmintic were significantly less frequently helminth-positive than cats that were not dewormed [12]. This is in agreement with the result obtained in our study: cats receiving no anthelmintic treatment were significantly more at risk for *T. cati* infestation than cats receiving one or two treatments per year, themselves being significantly more frequently infested than cats receiving more than 3 treatments per year.

The frequency of anti-flea treatment was also evaluated by multivariate analysis for flea infestation. Cats treated with an anti-flea product more than 4 times a year were significantly less at risk for flea infestation than cats receiving less than 3 treatments a year. Most of the commonly used anti-flea products have a persistent effectiveness of one month, but no data were analyzed in regards to the way treatments were applied, i.e. once every 3 months, or consecutively during the flea season, which is more probable. Cats treated more than 4 times a year probably receive better veterinary care than others, and regular anti-flea treatments may have an impact on flea infestation in the long term [61,62].

## Conclusions

The results of this study highlight the fact that both ecto- and endoparasites are still common in cats throughout Europe. Given the zoonotic consideration and the clinical importance, it is strongly advisable to promote effective and regular parasite control in cats, with adequate frequencies of treatment for both internal and external parasites.

## Competing interests

The author’s declare that they have no competing interests.

## Authors’ contributions

To include: All authors read and approved the final version of the manuscript.
